# The Pharmaceutical Uses of Saccharin

**Published:** 1887-02

**Authors:** 


THE PHARMACEUTICAL USES OF SACCHARIN.—“There seems to be good reason for believing that saccharin has no injurious physiological action when taken internally.

“Sir Henry Roscoe has stated that large quantities have been given to dogs, and one dog had as much fed to him daily as was equivalent in sweetening power to a pound of sugar, and did him no harm. Up to the present time it has not been known to produce any injurious effect upon the system, and will, therefore probably be more employed in pharmacy. Mr. C. J. S. Thomson, of Manchester, publishes, in the *Pharmaceutical Journal* for October 23, 1886, the following notes of some rough experiments made with a view of ascertaining how far the new sweetening principle (saccharin) may be of use in this way. He finds that one per cent. solution of saccharin in hot water, although very sweet to the taste, has a flavor distinctly different from that of cane-sugar. Ninety minims of this solution added to one containing four grains of sulphate of quinine quite marked the bitter taste of the alkaloid and rendered it palatable.

“Thirty minims of the same solution sweetened half a drachm of tincture of iron, and it required twenty minims to cover the salty taste of ten grains of bromide of potassium.”

The JOURNAL, some weeks since, published an article on this subject. Since then the Editor has received various communications from physicians asking where it can be obtained. It is learned that in a short time it will be put upon the market.

